# Antiviral Activity of Canine RIG-I against Canine Influenza Virus and Interactions between Canine RIG-I and CIV

**DOI:** 10.3390/v13102048

**Published:** 2021-10-12

**Authors:** Zhen Wang, Shaotang Ye, Congwen Yao, Ji Wang, Jianwei Mao, Liang Xu, Yongbo Liu, Cheng Fu, Gang Lu, Shoujun Li

**Affiliations:** 1 College of Veterinary Medicine, South China Agricultural University, Guangzhou 510642, China; wangzhenwz@stu.scau.edu.cn (Z.W.); yeshaotang@stu.scau.edu.cn (S.Y.); ycw1110@outlook.com (C.Y.); wangjiwj@stu.scau.edu.cn (J.W.); maojianwei@stu.scau.edu.cn (J.M.); liangxu@stu.scau.edu.cn (L.X.); lyb7572592692021@163.com (Y.L.); LG@scau.edu.cn (G.L.); 2 Guangdong Provincial Key Laboratory of Prevention and Control for Severe Clinical Animal Diseases, Guangzhou 510642, China; 3 Guangdong Technological Engineering Research Center for Pet, South China Agricultural University, Guangzhou 510642, China; 4 College of Animal Science & Technology, Zhongkai University of Agriculture and Engineering, Guangzhou 510642, China; fucheng@zhku.edu.cn

**Keywords:** canine RIG-I, antiviral, CIV, interaction

## Abstract

RIG-I functions as a virus sensor that induces a cellular antiviral response. Although it has been investigated in other species, there have been no further studies to date on canine RIG-I against canine influenza virus (CIV). In the present study, we cloned the RIG-I gene of beagle dogs and characterized its expression, subcellular localization, antiviral response, and interactions with CIV proteins. RIG-I was highly expressed and mainly localized in the cytoplasm, with low levels detected in the nucleus. The results revealed that overexpression of the CARD domain of RIG-I and knockdown of RIG-I showed its ability to activate the RLR pathway and induced the expression of downstream interferon-stimulated genes. Moreover, overexpression of canine RIG-I suppressed the replication of CIV. The association between RIG-I and CIV was evaluated with the luciferase assay and by indirect immunofluorescence and bimolecular fluorescence complementation analyses. The results showed that CIV nonstructural protein 1 (NS1) can strongly suppress the RIG-I–mediated innate immune response, and the novel interactions between CIV matrix proteins (M1 and M2) and canine RIG-I were disclosed. These findings provide a basis for investigating the antiviral mechanism of canine RIG-I against CIV, which can lead to effective strategies for preventing CIV infection in dogs.

## 1. Introduction

Canine influenza virus (CIV) is an eight segments single stranded virus of the *Orthomyxovirus* type A influenza virus family. CIV encodes at least 10 proteins, including PB2, PB1, PA, HA, NP, NA, M1, M2, NS1, NEP, and other proteins [1]. Equine-origin CIV H3N8 was first reported to be prevalent among dogs in 2004 in Florida, USA [2]. Subsequently, more different origins CIV strains were later detected in different countries, such as China and Korea [3,4,5]. Dogs might be a “mixing vessel” for the new influenza A virus reassortment in future [6]. Thus, further studies about canine innate immune response against CIV are crucial for new antiviral strategies investigation.

PAMPs are mediated by toll-like receptors (TLRs), retinoic acid-inducible gene I-like receptors (RLRs), NOD-like receptors (NLRs), and C-type lectin receptors (CLR). Retinoic acid-inducible gene I (RIG-I), a member of the RLR family, is an innate immunologic molecule that recognizes short (10–300 bp) triphosphorylated (5′ ppp) ssRNA and dsRNA. In noninfected cells and in the absence of the virus, RIG-I exists in a self-inhibitory conformation. After binding to the viral ligand, a conformational change exposes the N-terminal caspase activation and recruitment domain (CARD) of the receptor. This triggers adapter mitochondrial antiviral signaling (MAVS) [7,8] and induces the transcription of interferon (IFN) regulatory factor 3 (IRF-3) and IRF-7, which are involved in the type I IFN-mediated antiviral response. The binding of secreted IFN to its cognate receptor activates Janus kinase (JAK)/signal transducer and activator of transcription (STAT) signaling and expression of downstream IFN-stimulated genes (ISGs), leading to a higher-level response against the virus [9,10,11].

RIG-I detects many viruses including Newcastle disease, Sendai, influenza, rabies, and hepatitis C viruses [12]. Influenza A virus (IAV) is a negative-strand RNA virus that produces dsRNA or ssRNA, which are recognized by RIG-I in the host. Thus, IAV can trigger the classical antiviral innate immune response mediated by RLR signaling [13,14]. However, IAV has developed several tactics to evade host immune surveillance mechanisms that involve interactions between viral nonstructural protein 1 (NS1) and host RIG-I, tripartite motif-containing 25 (TRIM25), and MAVS [15,16,17].

RIG-I has been characterized in many species, but little is known about the role of canine RIG-I in canine influenza virus (CIV) infection. This was investigated in the present study by cloning canine RIG-I and examining its tissue distribution and subcellular localization; its interaction with viral proteins; and the antiviral response triggered by RIG-I. We demonstrate that activated RIG-I induces RLRs pathway signaling and the activation of ISGs as part of the host antiviral innate immune response. CIV non-structural protein 1 (NS1) has suppression effect to activated RLRs pathway. Moreover, RIG-I is available to interact with various CIV proteins, including CIV RNA polymerase subunits (PB2, PB1, and PA), nucleoprotein, NS1, and matrix proteins (M1 and M2), but further influenced mechanism needs to be clarified.

## 2. Materials and Methods

### 2.1. Cells, Viruses, and Animals

Human embryonic kidney (HEK) 293T cells and Madin–Darby canine kidney (MDCK) cells were cultured at 37 °C and 5% (*v*/*v*) CO_2_ in Dulbecco’s modified Eagle medium (DMEM; Biological Industries, Kibbutz Beit-Haemek, Israel) and 10% fetal bovine serum (Gibco, Grand Island, NY, USA). CIV H3N2 (A/canine/Guangdong/02/2011, C/GD/02) was propagated in specific pathogen-free chick embryos. Viruses and tissue samples from beagle dogs (Canis lupus familiaris) (Fuzhou Zhen and Experimental Animal Technology Development Company, Fuzhou, China) were stored at −80 °C.

### 2.2. RNA Extraction, cDNA Synthesis, and Cloning of the Canine RIG-I Gene

Total RNA was extracted from cells and tissues with RNAiso Plus (Takara Bio, Otsu, Japan) according to the manufacturer’s instructions. The amount of RNA in each sample was quantified with a NanoDrop One spectrophotometer (Thermo Fisher Scientific, Waltham, MA, USA). cDNA was prepared from total RNA using the HiScript III 1st Strand cDNA Synthesis Kit (Vazyme, Nanjing, China). The complete canine RIG-I cDNA was amplified from tissues of beagle dogs with the following protocol: 95 °C for 3 min; 35 cycles of 95 °C for 30 s, 67 °C for 30 s, and 72 °C for 3 min; and 72 °C for 10 min. PCR was performed with Phanta Super-Fidelity DNA Polymerase (Vazyme). The primers were designed based on the predicted canine RIG-I gene (XM_005626701.3) in the National Center for Biotechnology Information (NCBI) database; the sequences are shown in Supplementary Table S1.

### 2.3. Sequencing and Bioinformatic Analysis of Canine RIG-I

The PCR products were cloned into the pMD18-T vector (Takara Bio) and sequenced. The conserved functional domains of the full-length canine RIG-I cDNA sequence were analyzed using the SMART program (http://smart.embl-heidelberg.de/, 18 June 2018) and NCBI Conserved Domain Database (https://www.ncbi.nlm.nih.gov/cdd/, 18 June 2018). A phylogenic tree was constructed with the neighbor-joining method using MEGA v6.0 software (https://www.megasoftware.net/, 28 June 2018). Amino acid sequences were aligned with Clustal X v2.0 and edited using BoxShade server (https://embnet.vital-it.ch/software/BOX_form.html, 28 June 2018).

### 2.4. Plasmids, Reagents, and Antibodies

Full-length canine RIG-I (residues 1–925), the N-terminal CARD (residues 1–190), and CARD deletion fragment (RIG-IΔCARD, residues 191–925) were inserted into the mammalian expression plasmid p3 × CMV10-Flag and bimolecular fluorescence complementation (BiFC) vector Venus VC-155 (with split C-terminal fragments of green fluorescent protein [GFP]) by homologous recombination using the ClonExpress Ultra Cloning Kit (Vazyme). The sequences of CIV proteins were inserted into the pCMV-Myc plasmid and BiFC vector Venus VN-173 (with split N-terminal fragments of GFP). The following plasmids were constructed: Flag–RIG-I, Flag–RIG-I-CARD, Flag–RIG-IΔCARD, VC–RIG-I, VC–RIG-I-CARD, VC–RIG-IΔCARD, Myc-polymerase beta 2 (PB2), Myc-PB1, Myc-polymerase alpha (PA), Myc-hemagglutinin (HA), Myc-nucleoprotein (NP), Myc-neuraminidase (NA), Myc-NS1, Myc-matrix protein 1 (M1), Myc-M2, VN-PB2, VN-PB1, VN-PA, VN-HA, VN-NP, VN-NA, VN-NS1, VN-M1, and VN-M2. The primer sets used for cloning are listed in Supplementary Table S1. The RIG-I agonist used in this study was 5′ triphosphate hairpin RNA (3p-hpRNA) (1000 ng per transfection; InvivoGen, San Diego, CA, USA). Primary and secondary antibodies used in this study are listed in Supplementary Table S4.

### 2.5. Indirect Immunofluorescence Analysis (IFA)

To determine the subcellular localization of RIG-I and other proteins, HEK 293T or MDCK cells were transfected with plasmid or agonist when they reached 50% confluence. After 24 h, the cells were washed three times with phosphate-buffered saline and then fixed in cold 4% paraformaldehyde at room temperature for 10 min. For immunofluorescence labeling, cells were blocked for 10 min at room temperature (QuickBlock Blocking Buffer for Immunol Staining, Beyotime, Shanghai, China), then incubated overnight at 4 °C with the primary antibody and for 1 h at room temperature with the secondary antibody; nuclei were stained with 4′,6-diamidino-2-phenylindole (Beyotime) for 5 min. The fluorescence signal was observed with a confocal laser scanning microscope (SP8 TCS; Leica, Wetzlar, Germany) and three-dimensional image reconstruction was carried out with Las X software (Leica).

### 2.6. Luciferase Assay

IFN-β promoter (IFN-Luc), nuclear factor κB (NF-κB) response element (3 × PRDII-Luc), and IRF-3 response element (3 × PRDIII/I-Luc) reporter plasmids were constructed as previously described [18]. MDCK cells were seeded in a 12-well plate and cotransfected with 0.5 μg/well of reporter plasmid and 0.04 μg/well of pRL-TK plasmid along with various expression plasmids, RIG-I agonist, or empty vector (control). After 24 h, the cells were lysed and the luciferase activity in lysates was measured with the Dual Luciferase Reporter Assay Kit (Vazyme) according to the manufacturer’s instructions and normalized to the internal Renilla luciferase control. The assay was repeated three times.

### 2.7. Quantitative Real-Time PCR (qRT-PCR)

qRT-PCR was carried out on a LightCycler 480 (Roche, Basel, Switzerland) with ChamQ SYBR qPCR Master Mix (Vazyme) using the following program: 95 °C for 30 s, followed by 40 cycles of 95 °C for 10 s and 60 °C for 30 s. Samples were analyzed in triplicate and 3 independent experiments were performed. The mRNA expression levels of cytokines and ISGs relative to glyceraldehyde 3-phosphate dehydrogenase (GAPDH) were calculated with the 2^−∆∆Ct^ method and plotted as fold change compared to mock-transfected cells. Primers used for qRT-PCR were designed based on published sequences and are listed in Supplementary Table S2.

### 2.8. Virus Replication Kinetics

Mock- or plasmid-transfected MDCK cells were seeded in a 12-well plate and infected with CIV at a multiplicity of infection of 0.1. The supernatant at different time points (12, 24, 36, 48, 60, and 72 h) was collected and stored at −80 °C; 10-fold dilutions were prepared and 0.1 mL of diluted virus was added to MDCK cells grown in a 96-well plate. After 1 h of adsorption, fresh DMEM containing 2% fetal bovine serum was added to the wells. Viral replication was detected by enzyme-linked immunosorbent assay [18] at 48 h post infection and is expressed as median log10 (median tissue culture infectious dose [TCID50]/mL) [19].

### 2.9. Western Blotting

HEK 293T or MDCK cells were grown to 80% confluence and transfected with different gene expression plasmids or empty vector. Cells were infected with CIV (or left uninfected) 24 h after transfection. After 24 h, the cells were lysed using Minute Total Protein Extraction Kit for Animal Cultured Cells/Tissues (Invent Biotechnologies, Plymouth, MN, USA). Lysates were collected and centrifuged for 30 s. A total of 30 μg of each sample was separated by sodium dodecyl sulfate polyacrylamide gel electrophoresis and transferred to a nitrocellulose membrane that was blocked for 15 min with QuickBlock Blocking Buffer for Western Blot (Beyotime) and then incubated overnight at 4 °C with primary antibodies, followed by a 1-h incubation with secondary antibody. Protein bands were visualized by Odyssey Sa (Li-cor, Lincoln, NE, USA).

### 2.10. RNA Interference

Small interfering RNA (siRNA; psiRIG-I-1 and psiRIG-I-2) targeting the different fragments of canine RIG-I were prepared. The sequences are listed in Supplementary Table S3. The siRNAs and a scrambled negative control siRNA were synthesized by RiboBio (Guangzhou, China). HEK 293T or MDCK cells were transfected with the siRNAs using Lipo8000 (Beyotime). The expression of transcription factors, cytokines, and ISGs were evaluated by qRT-PCR.

### 2.11. BiFC Assay

HEK 293T cells were seeded in 12-well plates and transfected with plasmids containing C- and N-terminal fragments of GFP for 24 h. GFP fluorescence was observed with a confocal laser scanning microscope.

### 2.12. Statistical Analysis

Relative expression levels are presented as mean ± standard deviation. The statistical significance of differences was evaluated with the unpaired Student’s t test using Prism v6.0 software (GraphPad, La Jolla, CA, USA). (mean ± SD, *n* = 3, * *p* < 0.05, ** *p* < 0.01, *** *p* < 0.001).

### 2.13. Ethics Statement

All procedures involving animals were approved and monitored by the South China Agricultural University Experimental Animal Welfare Ethics Committee (permit number SYXK [YUE] 2014-0136).

## 3. Results

### 3.1. Bioinformatic Analysis of the Canine RIG-I Gene

Based on the predicted sequence of canine RIG-I in the NCBI database, we designed a primer pair to clone the full-length cDNA from beagle dog tissue. The results of agarose electrophoresis and sequencing indicated that the open reading frame (2778 bp) encoded a 925-amino acid protein; the sequence was deposited in GenBank (accession number MG835367) (Supplementary Figure S1C). By performing a BLAST search, we found that 1 nucleobase in canine RIG-I was a variant of the predicted sequence at amino acid position 291 (T291G). The conserved protein domains were predicted using SMART and NCBI CCD; canine RIG-I had 2 CARDs at the N terminus (residues 2–186), a DEXD box domain (residues 234–439), helicase domain (residues 562–723), and C-terminal regulatory domain (residues 807–913) (Supplementary Figure S1A,B). Multiple sequence alignment of the canine RIG-I with homologs in other species showed that the highest similarity was with feline RIG-I (89.0%). A phylogenetic tree constructed according to the complete RIG-I sequence showed a close genetic relationship between the two species (Supplementary Figure S1D).

### 3.2. Expression of Canine RIG-I in Beagle Dog Tissues

**Figure 1:**
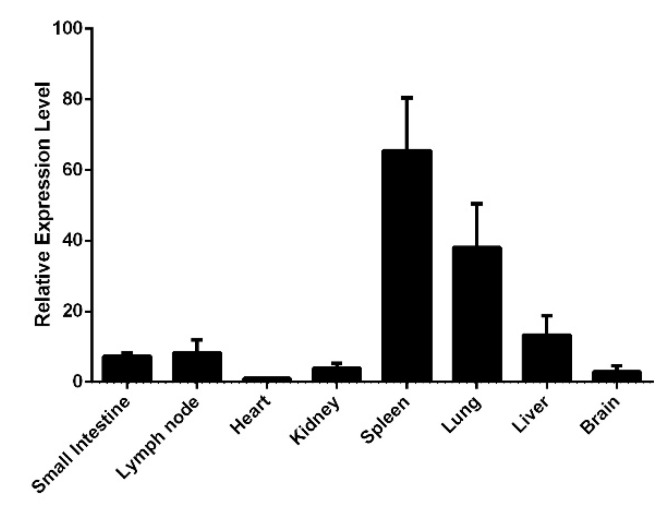
Distribution of canine RIG-I gene in different tissues of beagle dog. The expression level of canine RIG-I gene in each tissue is normalized by glyceraldehyde 3-phosphate dehydrogenase (GAPDH) mRNA and the heart tissue serves as a control. Samples were analyzed in triplicate and 3 independent experiments were performed. Error bars indicate standard deviations.

We examined the expression of the canine RIG-I gene in different tissues of beagle dog by qRT-PCR. RIG-I was broadly expressed in all eight tissues (including small intestine, intestinal lymph node, heart, kidney, spleen, lung, liver and brain) examined in this study, and was most highly expressed in the spleen and lung (Figure 1).

### 3.3. Subcellular Localization of Canine RIG-I Protein

**Figure 2:**
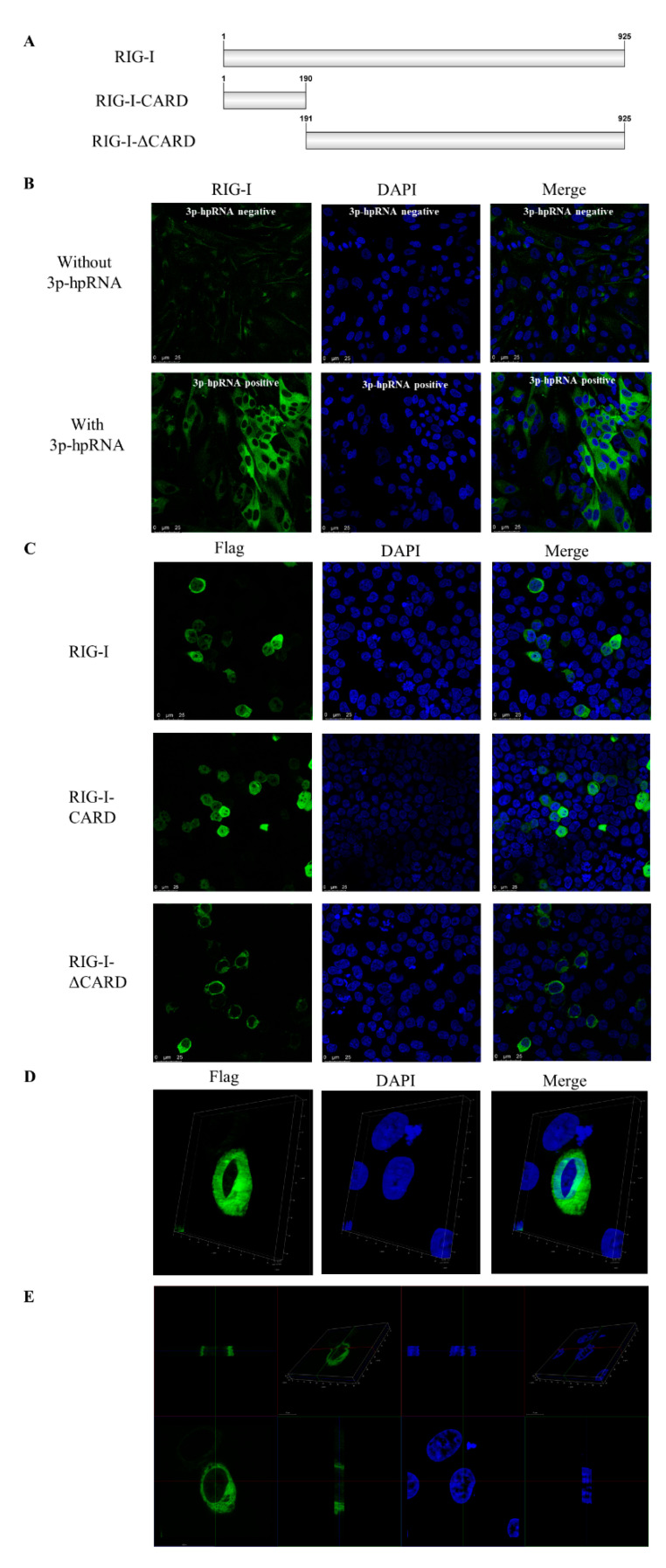
Subcellular localization of canine RIG-I in MDCK or HEK 293T cells. (**A**) Schematic diagram of plasmids of full-length canine RIG-I and truncated canine RIG-I. (**B**) MDCK cells were transfected with the RIG-I agonist 3p-hpRNA for 24 h. (**C**) HEK 293T cells were transfected with Flag–RIG-I, Flag–RIG-I-CARD, or Flag–RIG-IΔCARD for 24 h. (**D**,**E**) 3D reconstruction of HEK 293T cells transfected with Flag–RIG-I. The analysis was carried out by Las X software (Leica).

The subcellular localization of canine RIG-I was evaluated by IFA in MDCK cells transfected with the RIG-I agonist 3p-hpRNA for 24 h. RIG-I was mainly detected in the cytoplasm, with a low fluorescence signal observed in the nucleus (Figure 2A). In HEK 293T cells overexpressing Flag–RIG-I, the fusion protein was detected in the nucleus (Figure 2C,D). To identify the domain of RIG-I responsible for subcellular localization, Flag–RIG-I-CARD and Flag–RIG-IΔCARD were overexpressed in HEK 293T cells. Flag–RIG-IΔCARD showed similar localization to Flag–RIG-I, but Flag–RIG-I-CARD showed near-equal distribution in the cytoplasm and nucleus (Figure 2B). These results suggest that the CARD of canine RIG-I may contain a nuclear localization signal (NLS).

### 3.4. Antiviral Function of Canine RIG-I and Downstream ISGs

**Figure 3:**
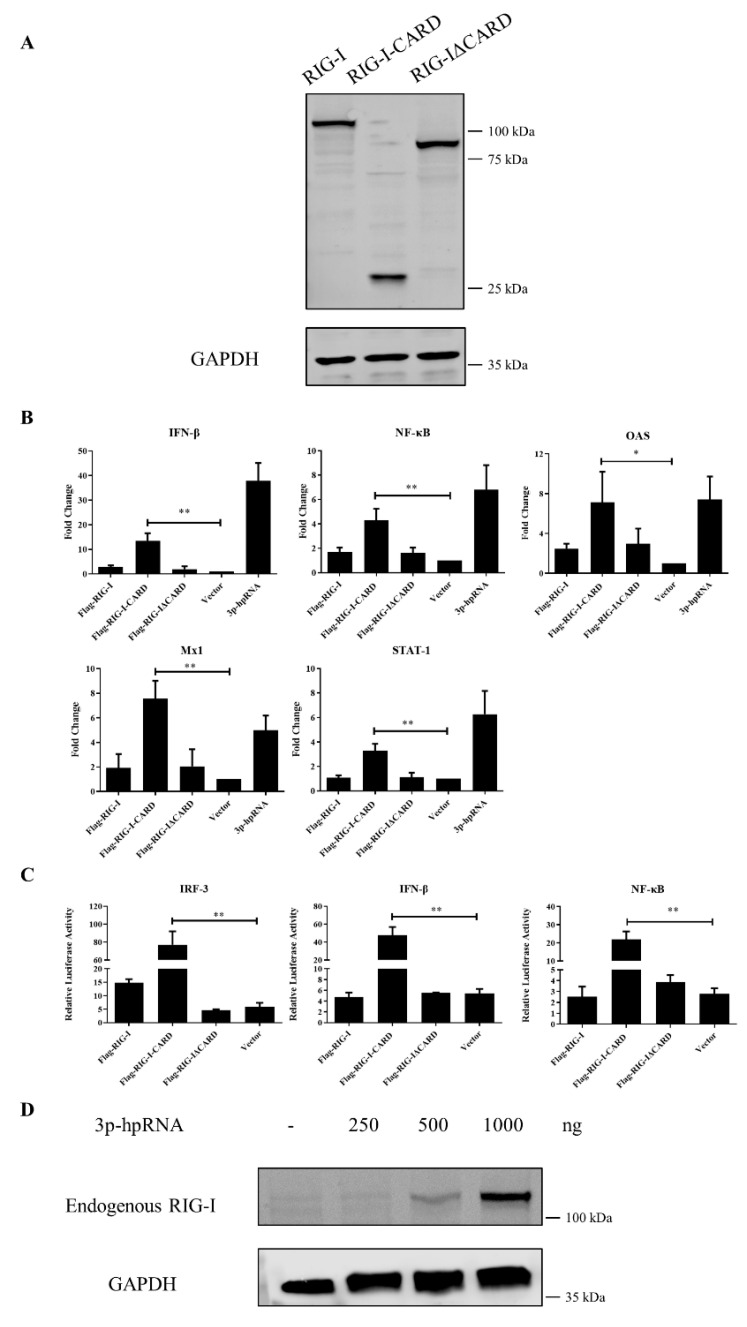
Gene expression in MDCK cells overexpressing full-length or partial forms of RIG-I or RIG-I agonist. (**A**) MDCK cells transfected with plasmids Flag–RIG-I, Flag–RIG-I-CARD or Flag–RIG-IΔCARD, respectively. The whole cells protein was obtained after 24 h. (**B**) Relative mRNA expression levels of NF-κB, IFN-β, Mx1, OAS, and STAT-1 were evaluated by qRT-PCR 24 h after transfection of Flag–RIG-I, Flag–RIG-I-CARD, Flag–RIG-IΔCARD, 3p-hpRNA, or empty vector. (**C**) Effect of overexpression of the various constructs on IRF-3, NF-κB, and IFN-β promoter activity as determined with the luciferase assays 24 h after transfection. Samples were analyzed in triplicate and three independent experiments were performed. * *p* < 0.05, ** *p* < 0.01 vs. empty vector control group. Error bars indicate standard deviation. (**D**) MDCK cells were transfected with indicated concentrations of RIG-I agonist and the whole cells protein was obtained after 24 h; the expression level of endogenous RIG-I was determined by western blotting with GAPDH used as a loading control.

In human, mouse, rat, pig, and other species, the CARD of RIG-I activates the antiviral response via a RIG-I–like signaling pathway. To investigate whether the CARD in canine RIG-I has a similar function, the whole cDNA or different fragments were cloned into the p3 × CMV10-Flag expression vector for overexpression in MDCK cells, and IFN induction was evaluated by qRT-PCR and the luciferase assay. The full-length canine RIG-I protein (Flag–RIG-I) and RIG-I lacking the CARD (Flag–RIG-IΔCARD) did not enhance the expression of downstream genes encoding transcription factors, cytokines, and ISGs in the absence of stimulation with the RIG-I agonist. However, these genes were significantly upregulated by overexpression of N-terminal CARD (Flag–RIG-I-CARD) and RIG-I agonist 3p-hpRNA. Notably, transcript levels of NF-κB were increased more than 4-fold, respectively (*p* < 0.01) whereas that of the cytokine IFN-β was increased more than 13-fold (*p* < 0.01). The expression of ISGs including MX dynamin-like GTPase 1 (Mx1; 7-fold, *p* < 0.01), oligoadenylate synthase (OAS; 7-fold, *p* < 0.05), and signal transducer and activator of transcription 1 (STAT-1; 3-fold, *p* < 0.01) was also enhanced (Figure 3B). Similarly, in the luciferase assay, CARD overexpression activated the IRF-3, NF-κB, and IFN-β gene promoters compared to the empty vector (*p* < 0.01) (Figure 3C). The western blotting results of MDCK cells transfected with the RIG-I agonist showed that endogenous RIG-I was strongly raised in a concentration-dependent manner (Figure 3D). Collectively, these results indicate that canine RIG-I can recognize the 3p-hpRNA and the CARD domain of canine RIG-I can induce a strong antiviral response.

### 3.5. RIG-I Deficiency Reduces the Antiviral Response

**Figure 4:**
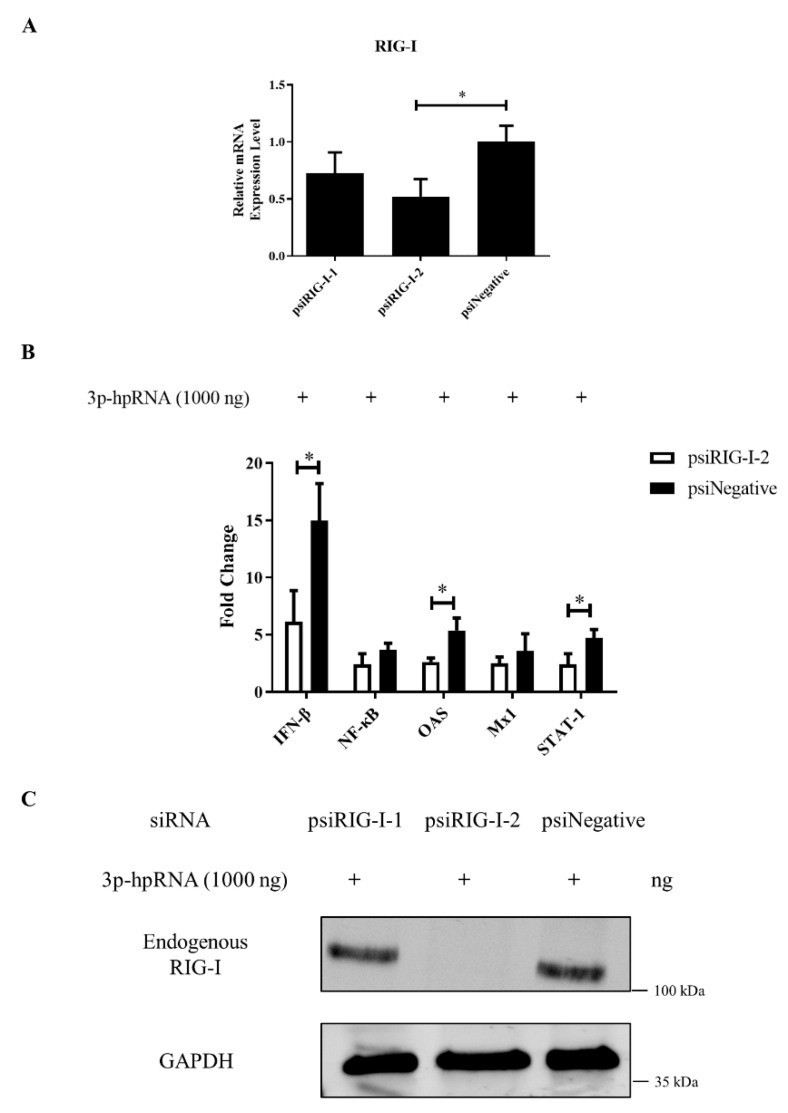
RIG-I knockdown suppresses the RIG-I–mediated antiviral response. (**A**) MDCK cells were transfected with 100 nmol of RIG-I siRNA or negative control. RIG-I mRNA expression level was evaluated by qRT-PCR 24 h after transfection. (**B**) MDCK cells were transfected with 100 nmol of psiRIG-I-2 and 1000 ng of the RIG-I agonist 3p-hpRNA to stimulate the antiviral response. NF-κB, IFN-β, Mx1, OAS, and STAT-1 mRNA expression levels were evaluated by qRT-PCR 24 h after transfection and were normalized to the level of GAPDH mRNA. Samples were analyzed in triplicate and three independent experiments were performed. * *p* < 0.05 vs. empty vector control group. Error bars indicate standard deviation. (**C**) MDCK cells were transfected with indicated 200 nmol siRNA and 1000 ng RIG-I agonist 3p-hpRNA together. The whole cells protein was obtained after 24 h; the expression level of endogenous RIG-I was determined by western blotting with GAPDH used as a loading control.

Two siRNAs targeting different parts of canine RIG-I were designed to examine the function of the protein in greater detail. The siRNA psiRIG-I-2 was more effective in knocking down the expression of endogenous RIG-I in MDCK cells (Figure 4A,C) and was used in subsequent experiments. RIG-I silencing significantly reduced the expression of IFN-β, OAS, and STAT-1, as determined by qRT-PCR (Figure 4B), whereas NF-κB and Mx1 were largely unaffected. These results suggest that canine RIG-I is required for activation of the antiviral innate immune response in dogs.

### 3.6. Canine RIG-I Inhibits CIV H3N2 Replication

**Figure 5:**
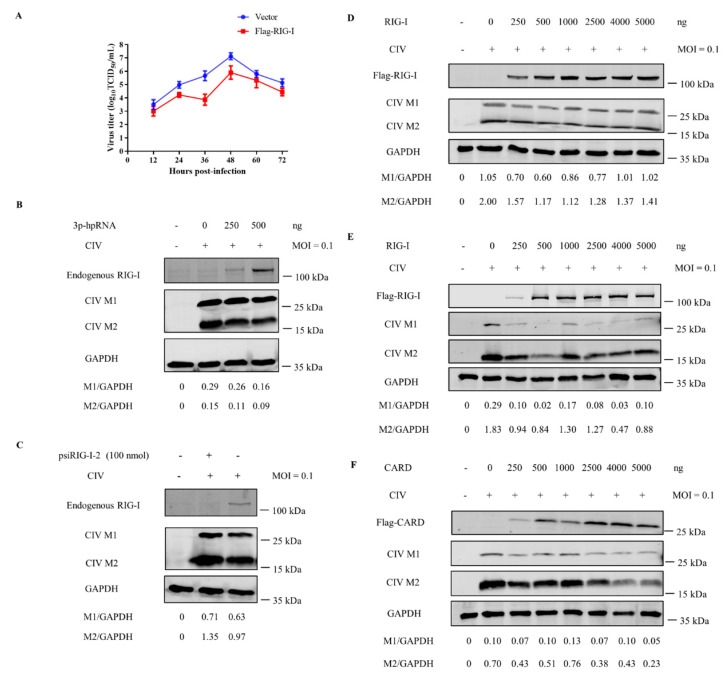
RIG-I inhibits the replication of CIV. (**A**) CIV H3N2 growth kinetics monitored over 72 h in MDCK cells transfected with Flag–RIG-I or empty vector. (**B**,**C**) MDCK cells were transfected with indicated concentrations of RIG-I agonist (**B**) or psiRIG-I-2 (**C**); after 24 h, cells were inoculated with CIV H3N2 at 0.1 multiplicity of infection (MOI) and the expression level of the endogenous RIG-I, viral M1 and M2 was determined by western blotting with GAPDH used as a loading control. (**D**) MDCK cells were transfected with indicated concentrations of Flag-RIG-I plasmid, after 24 h, cells were inoculated with CIV H3N2 at 0.1 MOI and the expression level of the Flag tagged RIG-I, viral M1 and M2 was determined by western blotting with GAPDH used as a loading control. (**E**,**F**) HEK 293T cells were transfected with Flag-RIG-I (**E**) or Flag-CARD (**F**) plasmids; after 24 h, cells were inoculated with CIV H3N2 at 0.1 MOI and the expression level of the Flag tagged RIG-I, Flag tagged CARD, viral M1 and M2 was determined by western blotting with GAPDH used as a loading control. Protein band intensity of endogenous RIG-I, Flag tagged RIG-I and CARD, viral M1 and M2, and GAPDH were used for western blotting quantification analysis.

RIG-I is known to activate antiviral responses by detecting IAV dsRNA or ssRNA. We examined whether canine RIG-I suppresses CIV via a similar mechanism. We first measured viral titers in MDCK cells overexpressing canine RIG-I at different time points after infection with CIV H3N2. The viral titer was lower in cells transfected with Flag–RIG-I as compared to the empty vector at each time point (Figure 5A). Furthermore, western blot analysis revealed that the viral M1 and M2 protein was downregulated in a dose-dependent manner in MDCK cells challenged with CIV after transfection with RIG-I agonist 3p-hpRNA or Flag-RIG-I vector (Figure 5B,D). Moreover, MDCK cells with canine RIG-I interference showed the higher yield of viral M1 and M2 protein than infected control (Figure 5C). Notably, with the higher transfection ration, CIV was also significant suppressed in HEK 293T cells transfected with the Flag-RIG-I or Flag–CARD plasmid (Figure 5E,F). Thus, canine RIG-I may effectively sense CIV H3N2 and inhibit its replication as part of the antiviral immune response in dogs.

### 3.7. Interaction between CIV and Canine RIG-I

**Figure 6:**
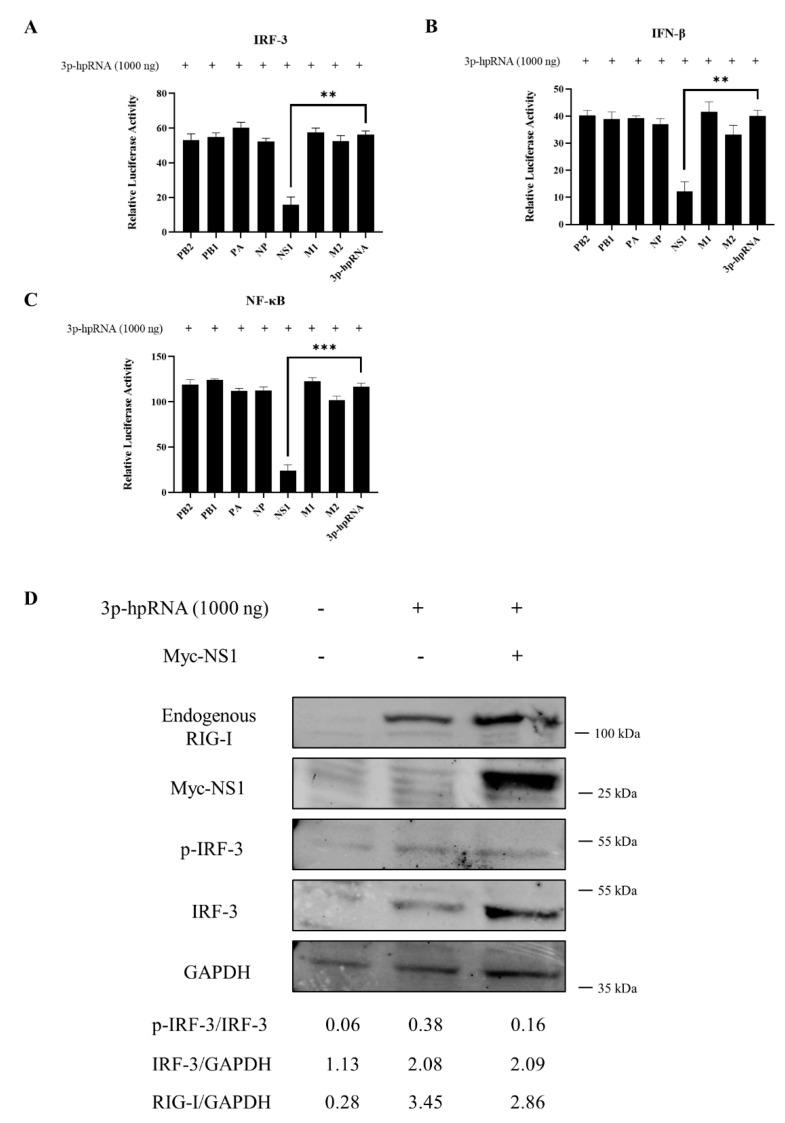
CIV NS1 impairs activation of RLR signaling in MDCK cells. (**A**) IRF-3 (**B**) IFN-β, and (**C**) NF-κB, promoter activity in the cells was evaluated 24 h after transfection with Myc-tagged CIV viral protein along with the 1000 ng RIG-I agonist 3p-hpRNA. Samples were analyzed in triplicate and three independent experiments were performed. ** *p* < 0.01, *** *p* < 0.001 vs. empty vector control group. Error bars indicate standard deviation. (**D**) MDCK cells were transfected with 1000 ng RIG-I agonist 3p-hpRNA along with or without Myc-NS1 plasmid; the whole cells protein was obtained for western blotting. Protein band intensity of endogenous RIG-I, IRF-3 and phosphorylated IRF-3 were used for western blotting quantification analysis.

**Figure 7:**
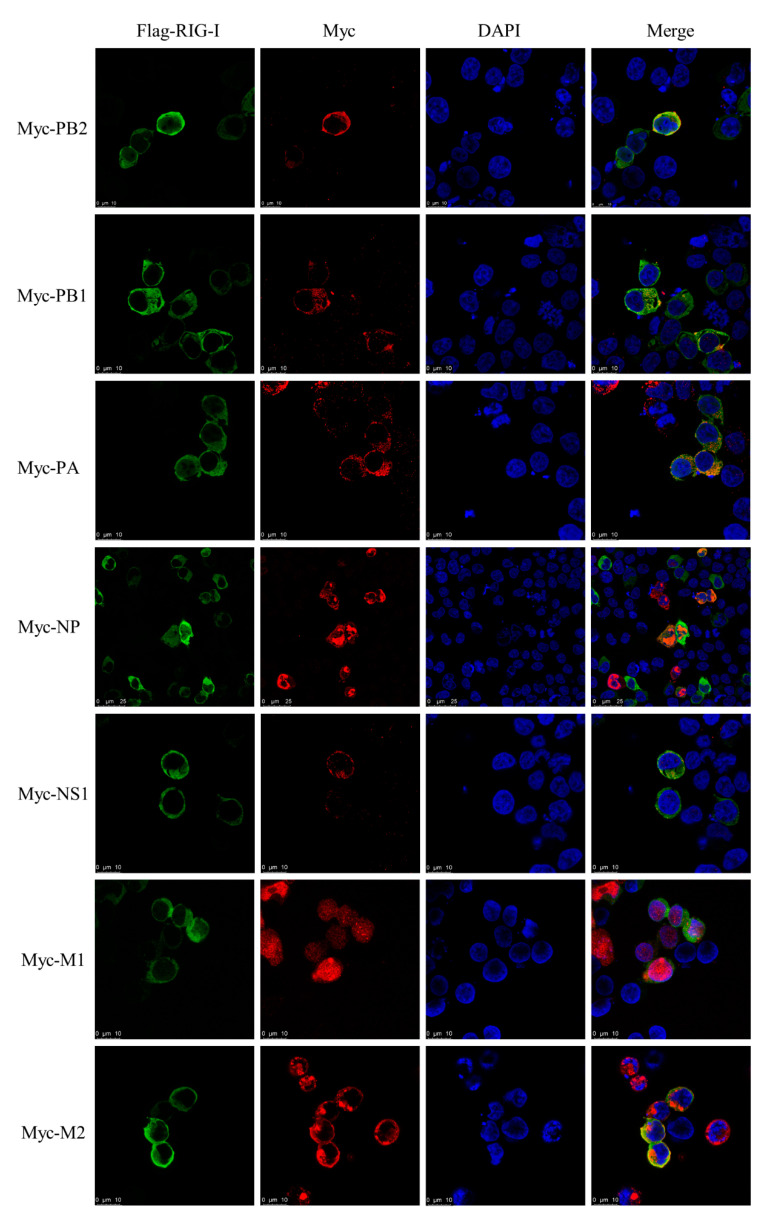
Interactions between CIV proteins and canine RIG-I observed by IFA in co-overexpression. IFA of canine RIG-I protein with Flag- or Myc-tagged CIV viral proteins (PB2, PB1, PA, NP, NS1, M1, and M2) in HEK 293T cells.

**Figure 8:**
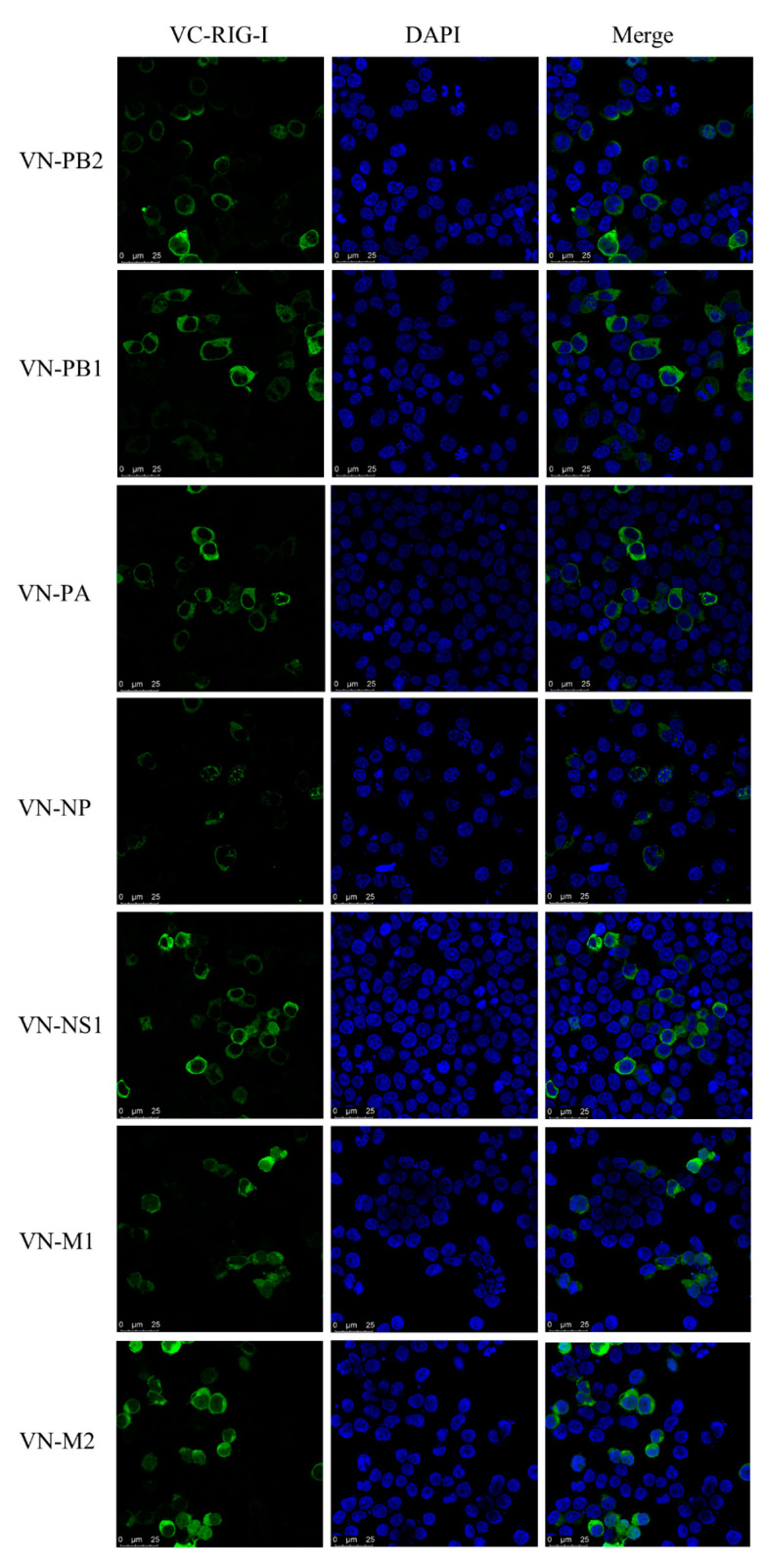
Interactions between CIV proteins and canine RIG-I detected by BiFC assay. BiFC analysis of the interaction between canine RIG-I protein (canine RIG-I with the C-terminal fragment of GFP) and CIV viral protein (PB2, PB1, PA, NP, NS1, M1, and M2 with the N-terminal fragment of GFP) in HEK 293T cells.

**Figure 9:**
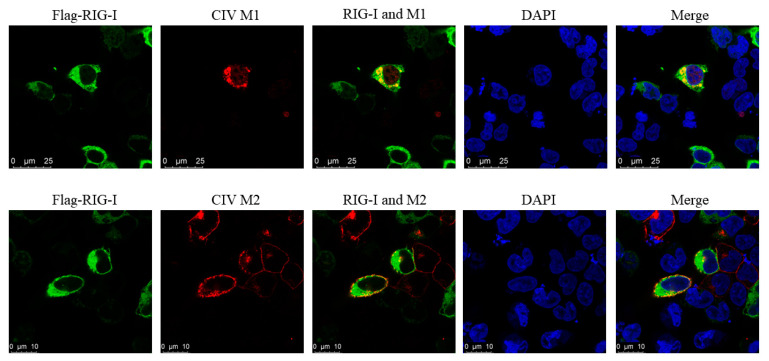
Interactions between CIV M1/M2 and canine RIG-I detected by IFA assay in CIV infection. HEK 293T cells was first transfected with Flag-RIG-I for 24 h, and they were secondarily infected with CIV for 24 h.

To investigate whether CIV NS1 could show the suppression to RLRs pathway, the luciferase assay and western blotting was carried out. CIV NS1 overexpression strongly suppressed the innate immune response (IRF-3, NF-κB, and IFN-β promoter activity) induced by the RIG-I agonist 3p-hpRNA in luciferase assays (Figure 6A–C). In western blotting, CIV NS1 showed the same ability to decrease the expression of the endogenous RIG-I and impair the phosphorylation of IRF-3 induced by RIG-I agonist 3p-hpRNA (Figure 6D). To further explore the protein interaction between CIV proteins and canine RIG-I, we carried out the co-overexpression in the IFA and BiFC assays. Notably, the results of IFA and the BiFC assays showed that RIG-I interacted with CIV ribonucleoprotein particle subunits (PB2, PB1, PA, and NP), NS1, and matrix proteins (M1 and M2). Moreover, PB2, PB1, PA, NP, NS1, M1, and M2 colocalized with RIG-I in the cytoplasm while NP colocalized with RIG-I in both the cytoplasm and nucleus (Figure 7 and Figure 8). Moreover, the IFA results of CIV infection also indicated the interactions between CIV M1/M2 and canine RIG-I (Figure 9). To keep the authenticity of IFA and BiFC assays, the negative control of IFA and BiFC assays were carried out and shown in supplementary materials (Figure S2).

## 4. Discussion

RLR is a PAMP that participates in the innate immune response. It is the main cytosolic viral RNA sensor and is highly conserved in eukaryotes. In this study, we cloned canine RIG-I from beagle dog tissue. An analysis of the 2778-bp open reading frame revealed 3 main conserved domains, tandem N-terminal CARDs, and DEXD box, helicase, and RIG-I C-terminal regulatory domains. The canine RIG-I sequence had a variant amino acid at position 291 (T291G) that was not present in the predicted sequence in the NCBI database, possibly due to individual differences among dogs. A phylogenetic analysis showed that canine RIG-I was most closely related to the homologous gene in cat [20,21,22].

We examined the tissue distribution and subcellular localization of canine RIG-I and found that it was broadly expressed in various tissues in beagle dogs, most prominently in the spleen and lung. In other species, RIG-I is highly expressed in immune organs [23,24,25] but canine RIG-I had low expression in lymph nodes, possibly due to the young age (and corresponding immaturity of the immune system) of the dogs used in this study. In MDCK cells stimulated with the RIG-I agonist 3p-hpRNA, RIG-I protein was mainly expressed in the cytoplasm as previously reported [26], although some nuclear localization was also observed. This was confirmed by IFA of HEK 293T cells transfected with the Flag–RIG-I plasmid. These results imply that as with the human protein, canine RIG-I protein shuttles between the cytoplasm and nucleus [27]. There is little information on the NLS or nuclear export sequence (NES) of canine RIG-I. To identify these parts of the protein, HEK 293T cells were transfected with plasmids harboring the full-length or partial RIG-I sequence. In cells expressing Flag–RIG-I-CARD, protein localization was near-equal in the cytoplasm and nucleus, but RIG-I lacking the CARD (Flag–RIG-IΔCARD) was detected only in the cytoplasm, suggesting that the NLS is found in the CARD. Additional experiments are needed to confirm the location of the NLS and identify the NES of RIG-I.

The short dsRNA and ssRNA of many viruses with 5′ ppp blunt ends are recognized by RIG-I [28]; 5′ ppp is required for RIG-I activation and is used by the host to discriminate self from viral RNA. In this study, 3p-hpRNA (an uncapped 5′ triphosphate hairpin RNA generated by in vitro transcription of an IAV sequence) was used as an agonist for canine RIG-I [29]. The endogenous canine RIG-I showed a strongly increasing trend under the stimulation in present study.

In the absence of viral infection, RIG-I is autorepressed through internal interactions between two domains (CARD2 and second CARD-helicase insertion domain [Hel2i]). When viral dsRNA or ssRNA binds to the C terminus, the CARD2–Hel2i interaction is disrupted by a conformational change and the exposed CARD binds to K63-linked polyubiquitin through ATP hydrolysis. TRIM25α mediates the ubiquitination of K63 in the CARD of RIG-I and induces its binding to MAVS [7,8], thereby activating a signal transduction cascade that leads to the translocation of the transcription factors IRF-3, IRF-7, and NF-κB to stimulate the expression of antiviral factors and induction of type-I IFN, which was confirmed in the present study by qRT-PCR. Moreover, IFN-β stimulates the expression of ISGs including IFN-induced transmembrane protein 1 (IFITM), Mx1, protein kinase R (PKR), and OAS, which function as effectors in the cellular response to virus infection [13,30,31]. In the current study, overexpressing the CARD of canine RIG-I and stimulating RIG-I with a specific agonist activated the RLR pathway and induced the expression of the transcription factors IRF-3 and NF-κB; the cytokine IFN-β; and ISGs Mx1, OAS, and STAT-1. Conversely, IRF-3, NF-κB, IFN-β, and Mx1 were downregulated by RIG-I knockdown. These results indicate that canine RIG-I plays an important role in regulating the antiviral response in dogs.

Elevated expression of IFN-β and ISGs suppresses IAV replication in the host during infection [13,31]. To determine whether canine RIG-I has this effect on CIV, we examined virus replication kinetics in MDCK cells and found that CIV titer was decreased at each post infection time point, especially at 36 h. This was accompanied by reduced expression of the CIV M1 and M2 protein in cells transfected with RIG-I agonist 3p-phRNA, full length canine RIG-I or truncated length of CARD domain. Furthermore, cells with interference of canine RIG-I showed a higher yield of viral protein compared to infected control group. Taken together, canine RIG-I can inhibit the replication of CIV, consistent with the activity of RIG-I in other species against IAVs.

IAVs have evolved a variety of strategies to evade host surveillance mechanisms. Previous studies have demonstrated that protein degradation, inhibited phosphorylation and ubiquitination in RLRs pathway results from the accumulation of multi-functional IAV NS1 [15,16,32]. In present study, the results of the luciferase assay and western blotting in MDCK cells showed that CIV NS1 protein suppressed the activation of the RLRs pathway, including down-regulating canine RIG-I and decreasing phosphorylation of IRF-3, which is in accordance with our previous finding that CIV NS1 inhibited the production of type I IFN induced by SEV [33]. Taken together, CIV NS1 does not directly down-regulate the expression of IRF-3, but it decreases the ratio of phosphorylated IRF-3 to inhibit the signaling transduction of RLRs pathway. CIV NS1 can reduce the protein expression level of canine RIG-I to restrain the activation of RLRs pathway. 

However, the interactions between canine RIG-I and CIV are not fully understood. This was addressed in our study by IFA and the BiFC assay. We found that RIG-I interacts with the PB2, PB1, PA, NP, NS1, M1, and M2 proteins of CIV. The subcellular location where canine RIG-I and CIV PB2 or NP interact differs from that reported in a previous study [34]; we found that RIG-I interacted with PB2 mainly in the cytoplasm and with NP in both the cytoplasm and nucleus, which may result from the shuttling activity of RIG-I. The interaction between RIG-I and M1 and M2, which function in virus budding and assembly, has not been previously described [35,36]. M2 also acts as a proton channel and activator of inflammation [36,37], and interactions between MAVS and M2 have been linked to autophagy and the innate immune response [38,39,40]. Additional studies are needed to clarify the functional significance of these novel interactions in the host response to viral infection.

In conclusion, this is the first report of the cloning and characterization of canine RIG-I, including the signaling pathway that is activated by RIG-I in the host response to CIV infection. We demonstrated that canine RIG-I stimulates the production of IFN and expression of downstream ISGs and showed that it interacts with multiple CIV proteins. These findings provide a basis for more detailed studies on the antiviral mechanisms of RIG-I that can potentially be exploited as a measure to protect dogs against CIV infection.

## Data Availability

The data that support the findings of this study are available from the corresponding author upon reasonable request.

## Supplementary Materials

The following are available online at https://www.mdpi.com/article/10.3390/v13102048/s1. Figure S1 Bioinformatic analysis of canine RIG-I gene; Figure S2: Negative control of IFA and BiFC assays; Table S1: Primer sets used for PCR in this study; Table S2: Primer pairs used for quantitative RT-PCR analysis; Table S3: Sequences of siRNA used in RNA interference; Table S4: Antibody used in this study.
