# Supplementary material for: Antiviral Activity of Canine RIG-I against Canine Influenza Virus and Interactions between Canine RIG-I and CIV
Source: Viruses. 2021 Oct 12;13(10):2048. doi: 10.3390/v13102048 (PMC8540569; doi:10.3390/v13102048)
Supplement: Supplementary file 1 [file viruses-13-02048-s001.zip › viruses-1395627-supplementary.pdf]

Table S1 Primer sets used for PCR in this study

Primer	Sequence of oligonucleotides (5′ → 3)
RIG-I-F	ATGACGGCCGAGGAGCGGCGAAA
RIG-I-R	TCATTCGGGTATTTCTGGAGAACCAAAGGGTATCTTC
RIG-I-CARD-F	ATGACGGCCGAGGAGC
RIG-I-CARD-R	TTTGTCCACAATCCACAGTTGACTGA
RIG-I-△CARD-F	ATGGATGTTGAACTGAAAGTTCTTGAGGAT
RIG-I-△CARD-R	TCATTCGGGTATTTCTGGAGAACCAA
CIV PB2-F	ATGGAGAGAATAAAAGAATTAAGAG
CIV PB2-R	TCACATTGATGGCCATCCGAATTCTTTTGGT
CIV PB1-F	ATGGATGTCAATCCGACTTTACTTT
CIV PB1-R	TCACTTTTTGCCGTCTGAGCTCT
CIV PA-F	ATGGAAGACTTTGTGCGACAAT
CIV PA-R	TCACTTTCAGTGCATGTGTG
CIV HA-F	ATGAAAACTGTTATTGCTTTAAGCTAC
CIV HA-R	TCACAATGCAAATGTTGCACC
CIV-NP-F	ATGGCGTCTCAAGGCACCAA
CIV-NP-R	TCACATTGTCATACTCTTCTACATTGTCTCCGAA
CIV-NA-F	ATGAACCCAAATCAGAAGATAATAGCAATAGGCTC
CIV-NA-R	TCACTATAGGCATGAAGTTGATATTCGCCCCAT
CIV-M1-F	ATGAGTCTTCTAACCGAGGTC

CIV-M1-R	TCACTTAAATCGCTGCAT
CIV-M2-F	ATGAGTCTTCTAACCGAGGTCGAA
CIV-M2--R	TTACTCCAGCTCTATGTTGACAAA
CIV-NS1-F	ATGGATTCCAACACTGTGTCAAGC
CIV-NS1-R	TCACAATTTCTGACTCAATTGTTCTCGC
Flag-homologous arm-F	GACGATGACAAGCTTGCGGCCGCG
Flag-homologous arm-R	CAGGGATGCCACCCGGGATCC
Myc-homologous arm-F	TGGCCATGGAGGCCCGAATTCAA
Myc-homologous arm-R	CCTCGAGAGATCTCGGTCGACAA
VC/VN-homologous arm-F	ACCGGACTCAGATCTGAATTCC
VC/VN-homologous arm-F	CTCCACCCTCGAGCGGGATCCC

Table S2 Primer pairs used for quantitative RT-PCR analysis

Primer	Sequence of oligonucleotides (5′ → 3)	Gene accession NO.
RIG-I-qF	GTTGCTGATGAAGGCATCGACATTG	XM_005626701
RIG-I-qR	CACTTGCTACCTCTTGCTCTTCCTC	
IRF-3-qF	CCAAGGATGAAGATGGAGACCTGTTC	XM_005616307
IRF-3-qR	GCGTGGTGAGTGTCGGCTTC	
NF-κB-qF	AACACTGGAAGCACGAATGACAGAG	NM_001003344
NF-κB-qR	CATGAGCCGCACCACACTGAG	
IFN-β-qF	CCAGTTCCAGAAGGAGGACA	NM_001135787
IFN-β-qR	TGTCCCAGGTGAAGTTTTCC	
Mx1-qF	GATTCGGACGAGGAGAAGAAGAAGAAG	NM_001003134
Mx1-qR	GATGTGGCTGGAGATGCGGTTG	
OAS-qF	ATCCCCTAACCCCTCTACACA	NM_001048131
OAS-qR	ATGCAAAATGCTCCACACAGG	
STAT-1-qF	AGTGGAAGCGGAGACAGCAGAG	XM_843260
STAT-1-qR	CAGCGACTACGGTGAACCAGTTC	
GAPDH-qF	AATTCCACGGCACAGTCAAGGC	NM_001003142
GAPDH-qR	ACAACATACTCAGCACCAGCATCAC	

Table S3 Sequences of siRNA used in RNA interference

Primer	Sequence of oligonucleotides (5′ → 3)
psiRIG-I-1	GGCTGAGAAGAACAATAAA
psiRIG-I-2	TGAGTAAGTTACACCCTAA

Table S4 Antibody used in this study

Antibody	Company	Host species	Catalog number
RIG-I	Millipore	Mouse	MABF297
Flag tag	GeneTex	Mouse	GTX82562
Myc tag	GenteTex	Rabbit	GTX115046
IAV M1	GeneTex	Rabbit	GTX125928
IAV M2	GenteTex	Rabbit	GTX125951
IRF-3	CST	Rabbit	#29047
Phospho-IRF-3 (Ser396)	CST	Rabbit	#11904
Alexa Fluor® 488	Abcam	Goat	AB150113
Alexa Fluor® 594	Abcam	Goat	AB150080

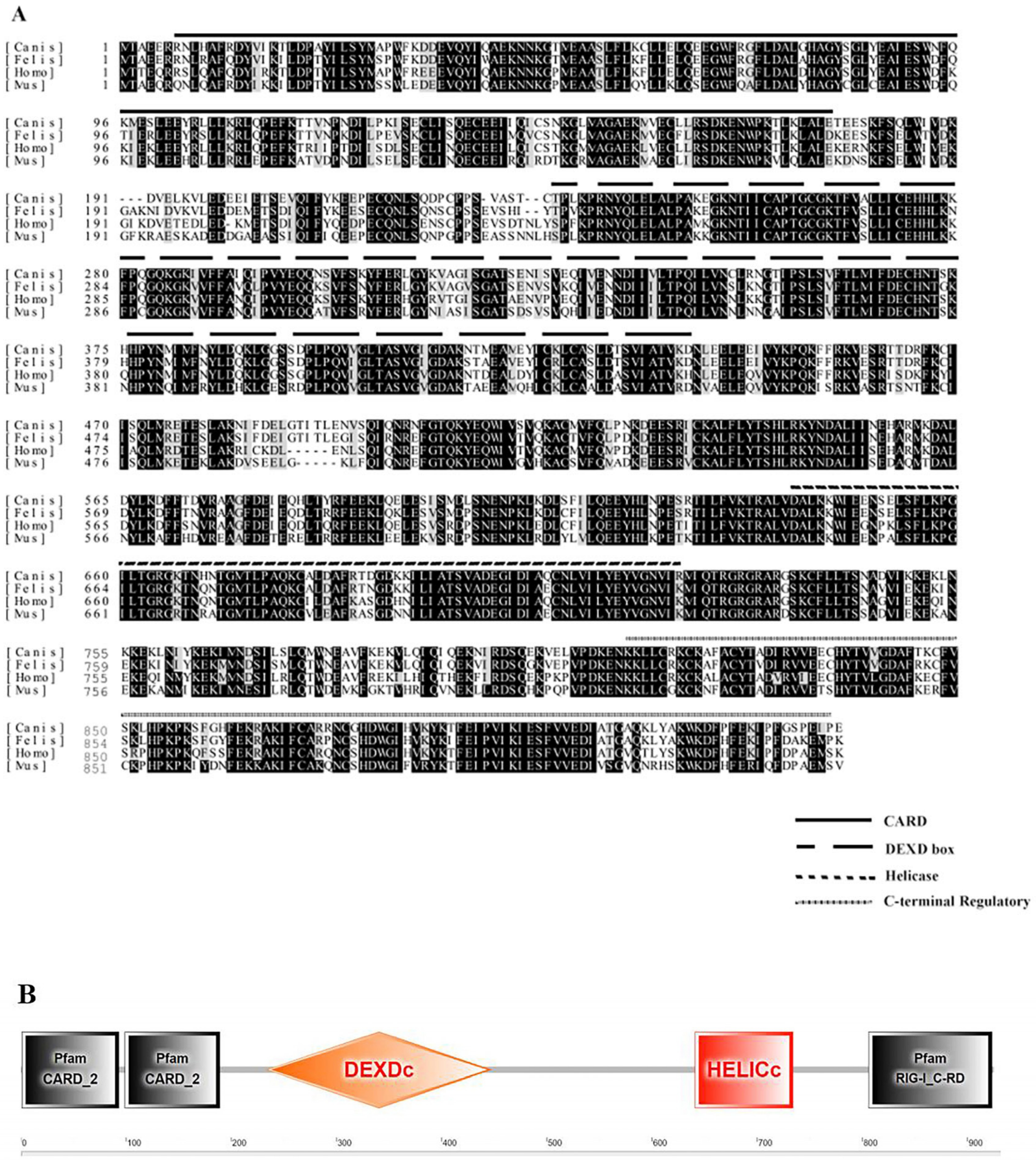
A
[Canis]
[Felis]
[Homo]
[Mus]
CARD
DEXD box
Helicase
C-terminal Regulatory
B
Pfam
CARD_2
Pfam
CARD_2
DEXDc
HELICc
Pfam
RIG-I_C-RD
0
100
200
300
400
500
600
700
800
900

C

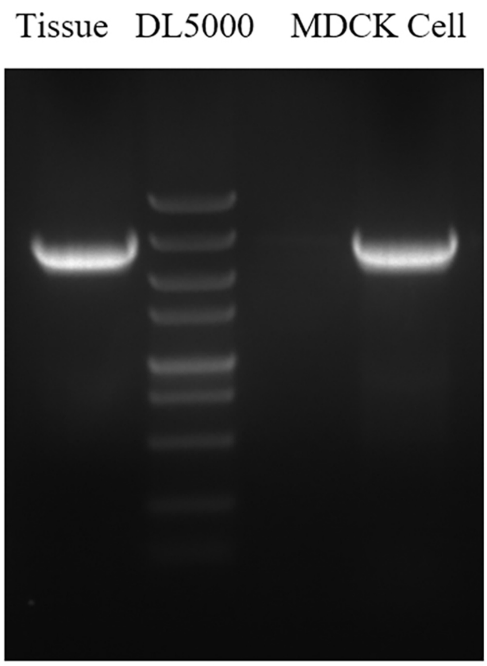

D

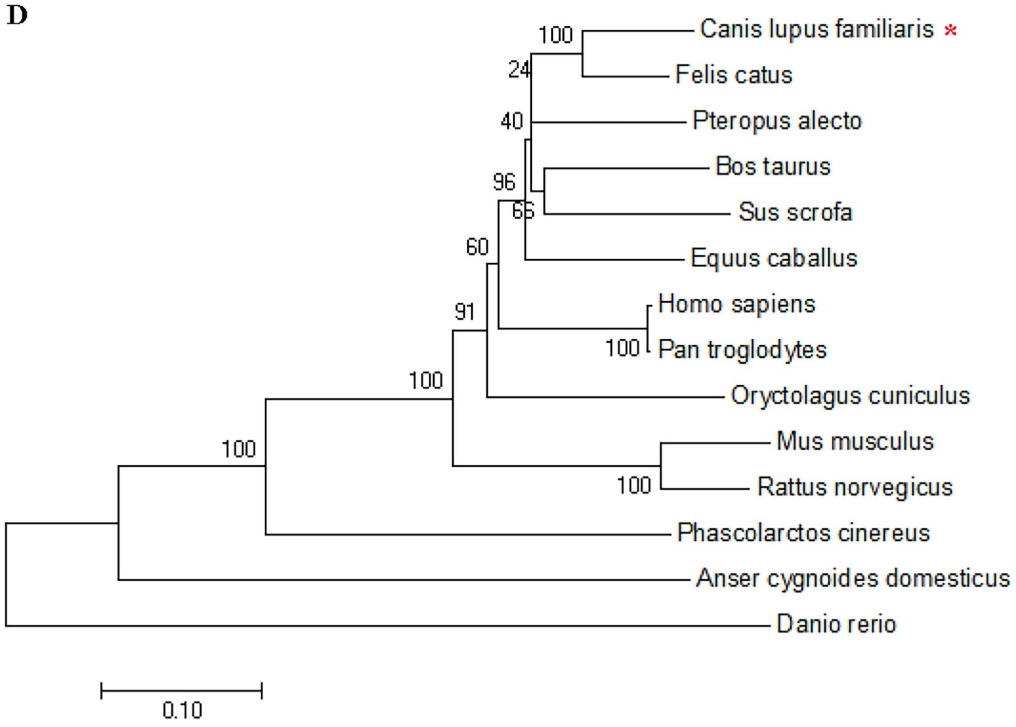

Figure S1 Bioinformatic analysis of canine RIG-I gene. (A) Amino acid multiple alignments of canine, feline, human, and mouse. Black considers as consensus. Alignment was performed using Clustal X program and edited with Boxshade. (B) The schematic representation of canine RIG-I. (C) The agarose electrophoresis of

canine RIG-I (Left track: canine RIG-I cloned from MDCK. Right track: canine RIG-I cloned from beagle dog). (D) A phylogenic tree based on the relationship of RIG-I between canine and other species. The scale bar is 0.1. GenBank accession nos.: *Canis lupus familiaris*: MG835367; *Felis catus*: XM_006939199.4; *Pteropus Alecto*: NM_001290158.1; *Bos taurus*: NM_001034527.2; *Sus scrofa*: NM_213804.2; *Equus caballus*: XM_005605049.3; *Homo sapiens*: NM_014314.3; *Pan troglodytes*: XM_001156662.4; *Oryctolagus cuniculus*: XM_002708040.3; *Mus musculus*: NM_172689.3; *Rattus norvegicus*: NM_001106645.1; *Phascolarctos cinereus*: XM_020976230.1; *Anser cygnoides domesticus*: NM_001311190.1; *Danio rerio*: NM_001306095.1.

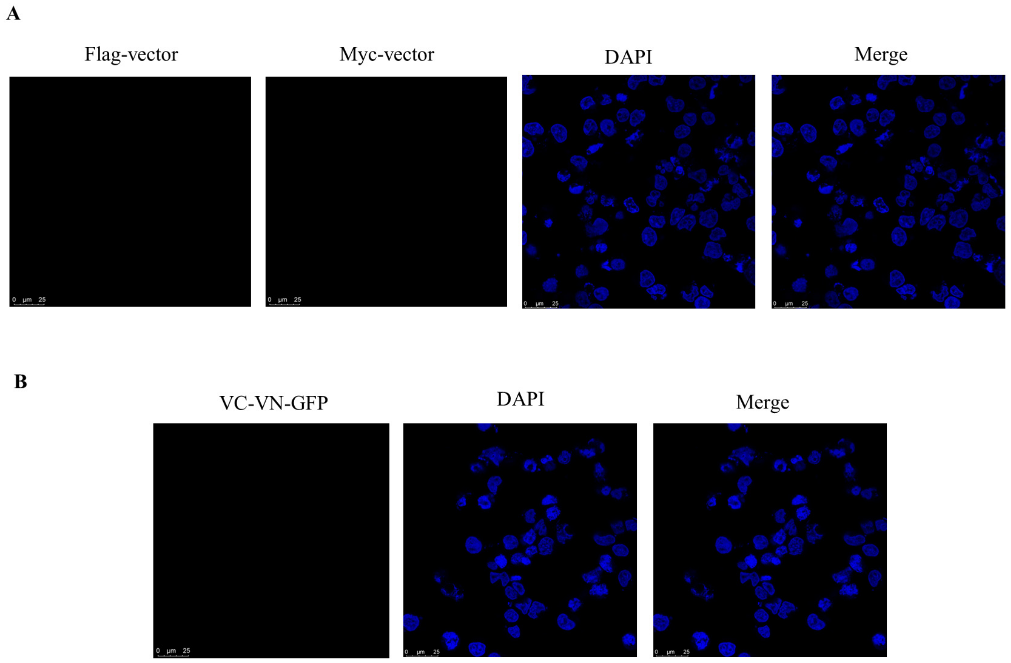

Figure S2 Negative control of IFA and BiFC assays. (A) HEK 293T cells were transfected with Flag tag vector and Myc tag vector together. The Flag tag and Myc tag primary antibody were used for IFA. (B) HEK 293T cells were transfected with

vector Venus VN-173 and vector Venus VN-173 together. After 24 h transfection, the sample was observed by confocal scanning microscopy.
